# Knowledge, attitudes, and perceptions of COVID-19 vaccine and refusal to receive COVID-19 vaccine among healthcare workers in northeastern Ethiopia

**DOI:** 10.1186/s12889-021-12362-8

**Published:** 2022-01-18

**Authors:** Metadel Adane, Ayechew Ademas, Helmut Kloos

**Affiliations:** 1 Department of Environmental Health, College of Medicine and Health Sciences, Wollo University, Dessie, Ethiopia; 2 Department of Epidemiology and Biostatistics, University of California, San Francisco, CA USA

**Keywords:** attitudes, COVID-19 vaccine, healthcare workers, knowledge, perceptions, vaccine refusal, Ethiopia

## Abstract

**Background:**

Major efforts are being made to control the spread and impacts of the coronavirus pandemic using vaccines. Ethiopia began on March 13, 2021, to vaccinate healthcare workers (HCWs) for COVID-19 with the AstraZeneca vaccine. However, willingness to be vaccinated depends to a large extent on factors beyond the availability of vaccines. This study aimed to determine the rate of intention to refuse COVID-19 vaccination   and associated factors among HCWs in northeastern Ethiopia. northeastern, Ethiopia.

**Method:**

An institution-based cross-sectional study  was employed among 404 HCWs in Dessie City, northeastern Ethiopia in May, 2021. Data were collected, checked, coded, entered into EpiData Version 4.6 and exported to Statistical Package of Social Sciences (SPSS) Version 25.0 for cleaning and analysis. The dependent variable was refuse to receive COVID-19 vaccination and the independent variables included socio-demographic factors, knowledge, attitudes and perceptions. A Binary logistic regression model was used to determine the association between vaccine refusal and the independent variables. From bivariate analysis, variables with *p*-values < 0.25 were retained for multivariable analysis. From multivariable analysis, variables with adjusted odds ratio (AOR), *p*-values <0.05 at 95% confidence interval (CI) were declared as factors significantly associated with refusal to be vaccinated among HCWs in Dessie City, northeastern Ethiopia.

**Results:**

The proportion of HCWs with overall good knowledge, good perception, and positive attitudes about COVID-19 vaccination were 62.5%, 60.5%, and 52.3%, respectively; 64.0% of the HCWs wanted to be vaccinated while 36.0% said that they would refuse to do so. Multivariable analysis identified negative attitudes (AOR: 3.057; 95%CI [1.860 - 5.026]) and poor perceptions (AOR: 4.73; 95%CI [2.911 - 7.684]) about COVID-19 vaccines were significantly associated with refusal to be vaccinated for COVID-19. Nearly half (46.9%) of the HCWs stated that vaccines could worsen any pre-existing medical conditions and 39.5% of them thought that vaccines could cause COVID-19 infections.

**Conclusion:**

The willingness of HCWs to get vaccinated against COVID-19 was relatively high among HCWs. Negative attitudes and poor perceptions towards the anticipated COVID-19 vaccination were significant factors to refuse to be vaccinated. Our findings may provide information for the management authorities and stakeholders to promote and improve attitudes, knowledge and perceptions towards COVID-19 vaccination uptake among HCWs.

## Background

COVID-19 infection rates are accelerating in Africa, including in Ethiopia. By October 24, 2021, Ethiopia had reported  362,088 COVID-19 cases and 6,347 deaths [1]. Many countries, including Ethiopia have implemented various strategies to control COVID-19, including declaring a state of emergency,  issuing restrictions on mass gathering, enforcing stay-at-home orders, and promoting the use of personal protective equipment [2]. As part of the worldwide strategy, efforts have been made to develop and distribute  vaccines. The COVAX program, backed by the World Health Organization (WHO) and other multilateral bodies, aims to supply 600 million doses to Africa, enough to vaccinate at least 20% of the population. However, by April 2021, only 18 million doses, representing 2% of all vaccine doses administered globally, had been administered by 41 African countries [3]. An additional 400 million doses of the Johnson & Johnson vaccine are scheduled to be shipped to the African Union starting in the third quarter of 2021 [4]. The first large shipment (2.2 million doses) of the AstraZeneca vaccine was received by Ethiopia in early March 2021 through the COVAX program. The country expected to receive an additional 5.4 million doses by May 2021 [5]. In addition, the African Vaccine Acquisition Trust (AVAT) announced the first monthly shipment of 108,000 doses of Johnson & Johnson vaccine to Ethiopia [6]. By April 2021, Ethiopia had administered only 430,000 doses of COVID vaccines, covering 0.2% of the country’s population with 2-shot vaccinations [7], but expected to cover 20% of the population by the end of 2021 [5, 8]. As of October 26, 2021, while more than 3.84 billion people worldwide had received one dose of a COVID-19 vaccine (equal to about 50% of the world population), 24 African countries had vaccinated less than 3% of their populations and only 0.9% of Ethiopia’s population had been fully vaccinated [9].

Ethiopia began on March 13, 2021, to vaccinate HCWs for COVID-19 with the AstraZeneca vaccine. Healthcare workers include medical doctors, laboratory technicians, nurses, midwives, pharmacists, radiographers, anesthesiologists, public health and environmental health officers, any any other professionals including non-medical auxiliary staff who work in a healthcare facility. Non-medical auxiliary staff are    other than health professionals such as  financial workers, human resource workers, janitors, card room and documentation workers, porters, guardians and others.

Elderly people above the age of 60 and those above 50 years who have  chronic diseases are also prioritized by the Ethiopian Ministry of Health. The launch of the vaccine program was attended by the WHO country representative Boueri Hama Sambo, who urged "communities and community leaders to build trust" as well as "demand for the vaccine" and to "promptly address "misinformation” [8]. During that event, one health worker was not convinced, saying "I don't believe in this vaccine. The virus is a sign of God’s wrath upon us, so I prefer to pray” [8]. Negative attitudes and mistrust towards COVID-19 vaccines are major barriers to increasing vaccine coverage worldwide [10].

Despite the scarcity of COVID-19 vaccines in Africa, several countries including Sierra Leone and Malawi have discarded large numbers of vaccines doses that expired before they were used due to low demand in the population, a situation also anticipated in Uganda [11]. In addition to the low accessibility of COVID-19 vaccines and hesitancy to be vaccinated for COVID-19, a preference for traditional medicines may constitute another barrier to vaccine acceptance in African countries, including Ethiopia. Indigenous medicines have been used to treat COVID-19 in Tanzania and Madagascar without meeting safety and efficacy standards [12]. The use of these plant products to treat COVID-19 carries safety risks and may hinder use of  scientifically proven COVID-19 vaccinations. A study in Nepal revealed that the use of medicinal plants has increased since the outbreak of the COVID-19 pandemic [13]. The use of traditional medicine for COVID-19 and its impact on vaccination rates has not been investigated in Ethiopia. However, it is known that some plant extracts have traditionally been used to treat influenza [14], and people may prefer using them rather than  accepting COVID-19 vaccines in the absence of diagnostic tests.

Willingness to receive a COVID-19 vaccine is a challenge in many countries [15]. COVID-19 vaccine acceptance rates in the general population were highest in Vietnam (98%), India (91%), China (91%), Denmark (87%), and South Korea (87%) and lowest in Serbia (38%), Croatia (41%), France (44%), Lebanon (44%), and Paraguay (51%) [16]. High hesitancy rates were also reported worldwide among HCWs, who play a central role in reducing the burden of the pandemic through their role in modeling preventive behavior and administering vaccinations. A review of 35 studies revealed that vaccination hesitancy rates varied from 4.3% to 72% worldwide [17]. Major concerns of health workers were vaccine safety, efficacy, and potential side effects. Higher socioeconomic status, directly working with patients, perceived risk and fear of COVID-19, and a history of influenza vaccination were associated with higher vaccine uptake [17]**.**

Reluctance of HCWs to accept COVID-19 vaccination may not only increase the risk of virus transmission to their patients but also reduce the likelihood that HCWs  will encourage patients to be vaccinated [17]. There is no documented evidence regarding attitudes and perceptions of Ethiopian health workers towards COVID-19 vaccination. This study examines knowledge, attitudes, perceptions towards the COVID-19 vaccine, level of refusal and associated factors of COVID-19 vaccination among HCWs in Dessie City, Ethiopia.

## Methods

### Study design, period and setting

This institution-based cross-sectional study  was conducted in Dessie City in May, 2021. Dessie is the capital of South Wollo Zone, located about 400 km from Addis Ababa. The city is located at an altitude of 2,470 to 2,550 meters. According to Dessie City administration health office, the city has 8 health centers, 2 government hospitals, 3 private hospitals and 15 clinics.

### Source population, inclusion and exclusion criteria

The source population for the study included all HCWs in Dessie City**.** The study population was all HCWs in the selected health facilities. All HCWs present at the selected health institutions during the survey were included in the study. Those who were absent (on sick leave, annual leave, or maternity leave) were excluded and not considered in sampling procedure (interval calculation) based on payroll lists.

### Sample size determination and sampling procedure

A total of 404 study participants were identified by using the single population proportion formula and considering the following assumptions:

$$n=\frac{{\left({z}_{a/2}\right)}^2\ast p\left(1-p\right)}{d^2}$$

where: ***n***: is the minimum sample size required, ***Z***_***α/2***_ is the standard normal variable at (1-α) % confidence level (α is 0.05 with 95% CI, Z_α/2_ = 1.96), *P* is an estimate of the attitude towards COVID-19 vaccine (50%) and ***d*** is the margin of error (5%). Based on these assumptions, 384 individuals were estimated. Then 5% of the sample size contingency was added to minimize errors arising from the likelihood of non-compliance, giving a final sample size of 404.

To select study subjects, 11 health institutions (2 government hospitals, 2 private hospitals, 4 health centers, and 3 private clinics) were randomly selected. For the selected healthcare facilities, study subjects’ proportional allocation was made based on their number regardless of the profession. Then each institution's payroll for all professions and systematic random sampling was used to identify study participants. The first participant was selected by the simple lottery method. Professions represented in the study included medical doctors, laboratory technicians, nurses, midwives, ophthalmologists, pharmacists, public health officers, radiographers, environmental health specialists, anesthesiologists, porters, medical record workers, administrative staff, cleaners, laundry workers, and guards.

### Outcome measurement and explanatory variables

The independent variables were socio-demographic factors, knowledge, attitudes, and perceptions and the dependent variable was refusalof to receive COVID-19 vaccination. To compute the three outcome variables of knowledge (good or poor), attitude (positive or negative), and perception (good or poor), we used the mean score of responses to 9 questions about knowledge and perceptions and 8 questions about attitudes. A score of **1** was assigned for poor knowledge, negative attitude and poor perception and a score of **2** for good knowledge, positive attitude and good perception. Scores above the mean value for each category were considered as good knowledge, positive attitude, and good perception. In addition, refusal to receive the COVID-19 vaccine was correlated as an outcome variable with socio-demographic, knowledge, attitude, and perception as independent variables.

### Data collection and quality assurance

The data were collected using a structured questionnaire adapted from relevant literature. Data were collected both through a self-administered questionnaire and interviews with selected professionals**.** Ten nurses with BSc degrees and 5 environmental health professionals with BSc degrees were employed as data collectors and supervisors. To ensure the acquisition of reliable data, both data collectors and supervisors were trained by the principal investigator for one day on the objectives of the study, the content of the questionnaire, ethical issues, and methods of data collection. We also pre-tested the questionnaire on 5% of the sample size among HCWs in nearby Haik Town before actual data collection and made adjustments in the questionnaire where necessary. The collected data were checked daily by the supervisors for completeness. To ensure accurate and reliable data, the reliability of the questionnaire was assessed using the Cronbach alpha test, giving values of 0.82, 0.79, and 0.87 for knowledge, attitude and perception questions, respectively. We also assessed the validity of this questionnaire by recruiting  experts to evaluate the content of the questionnaire using face validity, resulting in the experts agreeing that the test was a valid measure of the concept being measured. This means that they evaluated whether each of the measured items matched any given domain of the concept.

### Data management and statistical analysis

The collected data were checked, coded, and entered into EpiData Version 4.6 and exported to Statistical Package for Social Sciences (SPSS) Version 25.0 for data cleaning and analysis. Descriptive statistics, such as frequency distribution (n) and proportions (%) were computed. We ran a logistic regression analysis using socio-demographic, knowledge, attitudes and perceptions as independent variables and refusal of vaccination as the dependent variable. First, bivariate logistic regression analysis (crude odds ratio [COR] at 95% confidence interval [CI]) was performed and then variables with *p<* 0.25 were retained for multivariablelogistic regression analysis.

 From the multivariable analysis, variables with a significance level of *p<* 0.05 with adjusted odds ratio (AOR) at 95% CI were taken as statistically significant factors that were independently associated with the refusal of vaccination.

Model goodness of fit was checked using the Hosmer and Lemeshow test; *P-*values greater than 0.05 showed a value of 0.27, indicating the model was fit. Multi-collinearity between independent variables was also checked using the standard error of the coefficient of the model with a cut off value of 2. A maximum standard error value of 0.683 was reported from our model indicating the absence of multi-collinearity between independent variables.

## Results

### Socio-demographic characteristics of respondents

Of the 404 study participant HCWs, the response rate was 97.0%. The mean age of the respondents was 34.37 years (standard deviation [SD] = ± 7.785) and 198 were male (50.5%) and 194 female (49.5%). The study participants included 34 (8.7%) medical doctors, 46 (11.7%) laboratory technicians, 130 (33.2%) nurses and midwives, 50 (12.7%) pharmacists, 18 (4.6%) radiographers, 21 (5.4%) anesthesiologists, 36 (9.2%) public health officers and environmental health officers, and 57 (14.5%) non-medical auxiliary staff. The majority were university degree holders 257 (65.6%), 315 (80.4%) did not have health insurance, and more than one-third 157(40.1%) had received training or orientation about COVID-19 vaccination. The great majority (91.3%) of respondents did not have any chronic condition while 3.6% had hypertension, 2.3% asthma, 1.8% diabetes, and 0.5% each had HIV/AIDS and/or kidney disease (Table 1).

**Table 1:** Socio-demographic characteristics among healthcare workers in Dessie City, Ethiopia, May 2021

Questions	Frequency (*n*)	Percentage (*%*)
**Sex**		
Male	198	50.5
Female	194	49.5
**Age (years)**		
20-30	157	40.1
31-40	156	39.8
41-50	16	16.8
51-60	13	3.3
**Education status**		
Primary level	7	1.8
Secondary level	9	2.3
Diploma	119	30.3
University degree	257	65.6
**Profession**		
Medical doctor	34	8.7
Laboratory technician	46	11.7
Nurse and midwive	130	33.2
Pharmacist	50	12.7
Radiographer	18	4.6
Anesthesiologist	21	5.4
Public health officer	36	9.2
Nonmedical auxiliary staff	57	14.5
**Religion**		
Orthodox Christian	213	54.3
Protestant	29	7.4
Muslim	150	38.3
**Marital status**		
Single	144	36.7
Married	214	54.6
Divorced, widowed or separated	34	8.6
**Where did you grow up?**		
Rural	151	38.5
Semi-urban	77	19.6
Urban	164	41.8
**Household size (persons)**		
≤5	277	70.7
>5	115	29.3
** Years worked in a healthcare facility**		
10 or less	306	78.1
>10	86	21.9
**Do you have health insurance?**		
No	315	80.4
Yes	77	19.6
**Has anyone in your family or of your colleagues or friends had COVID-19?**		
Yes	129	32.9
No	263	67.1
**Have you been tested for COVID-19?**		
Yes	132	33.7
No	260	66.3
**If “yes”, were you positive? (*N*** **= 132)**		
Yes	25	18.9
No	107	81.1
**Do you have any chronic diseases?**		
Yes	34	8.7
No	358	91.3
**Smoking status**		
Current smoker	12	3.1
Ex-smoker	33	8.4
Never smoked	347	88.5
**Have you received any training or orientation about COVID-19 vaccination?**		
Yes	157	40.1
No	235	59.9
**How many times have you received training/orientation?**		
Once	116	29.6
More than once	41	10.4
**Source of information about COVID-19 vaccine (multiple responses possible)**		
Television	326	83.2
Internet	263	67.1
Radio	230	58.7
Scientific literature	159	40.6
Relatives and friends	127	32.4

### HCWs knowledge about COVID-19 vaccines

The mean score of the knowledge was 14.97, with a standard deviation of 1.881. The overall good knowledge rate of the HCWs about COVID-19 and its vaccine was 62.5% with 95% CI (57.4-66.8%). Of those who had good knowledge of COVID-19 and its vaccine, 171 (69.79%) were planning to be vaccinated as soon as a vaccine becomes available. One hundred twenty-eight (64.65%) males and 117 (60.3%) females had good knowledge whereas 70 (35.35%) males and 77 (39.69%) females had poor knowledge about COVID-19 and its vaccines. Similarly, 20 (58.8%) doctors, 31 (67.4%) laboratory technicians, 84 (64.6%) nurses, and midwives and 31 (54.38%) non-medical axillary staff had good knowledge. Among those who had a chronic disease, 22 (64.7%) had good knowledge. The majority (340, 86.7%) responded that COVID-19 is a serious disease and 329 (83.9%) stated that it could result in many health complications. One hundred fifty (38.3%) HCWs considered the differences in the effectiveness among  the Pfizer, Moderna, and Astra Zeneca COVID-19 vaccines to be large. More than three-quarters (77.6%) of the HCWs considered the major mode of COVID-19 transmission to be touching contaminated surfaces and touching one’s eyes, nose, and mouth, followed by shaking hands, hugging, and kissing (70.4%) and inhaling the virus (69.9%) (Table 2).

**Table 2:** Knowledge about  COVID-19 and  COVID-19 vaccines among healthcare workers in Dessie City, Ethiopia, May 2021

Questions	Frequency (*n*)	Percentage (*%*)
**Is COVID-19 a serious disease?** *		
Yes	340	86.7
No	29	7.4
I don't know ^δ^	23	5.9
Do you know that COVID-19 can result in complications? *		
Yes	329	83.9
No	63	16.1
Can vaccines effectively prevent COVID-19? *		
Yes	161	41.1
No	112	28.6
I don't know ^δ^	119	30.4
Can COVID-19 be acquired after full vaccination? *		
Yes	132	33.7
No	111	28.3
I don’t know ^δ^	149	38.0
Do you know where you can be vaccinated when a COVID-19 vaccine becomes available? *		
Yes	255	65.1
No	137	34.9
Are there large differences in the effectiveness of the Pfizer, Moderna, and Astra Zeneca COVID-19 vaccines? *		
Yes	150	38.3
No	242	61.7
**Does the effectiveness of the major COVID-19 vaccines vary between about 50% and 95%? (*N*** **= 150)**		
Yes	103	68.7
No	17	13.3
I don’t know	30	20.0
Do COVID-19 vaccines have side effects? *		
Yes	279	71.2
No	48	12.2
I don’t know ^δ^	65	16.6
Are older people and chronic disease patients most likely to experience severe illness and death from COVID-19 infection?*		
Yes	354	90.3
No	38	9.7
Are HCWs more vulnerable to COVID-19 infection than the general public? *		
Yes	340	86.7
No	25	6.4
I don’t know ^δ^	27	6.9
**Knowledge of HCWs about the mode of transmission of COVID-19 (multiple responses possible)**		
Poor hygiene	218	55.6
Inhalation of the virus	274	69.9
Touching contaminated surfaces, eyes, nose or mouth	304	77.6
Shaking hands, hugging, and kissing	276	70.4

*These questions are used to compute the overall score of knowledge

^δ^*The third choice “I don’t know” in some questions was included in the “No” category for the analysis*

**Fig. 1:**
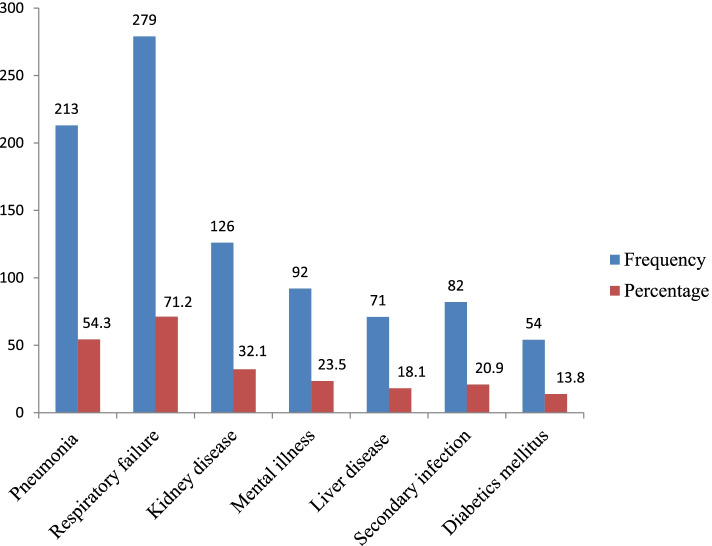
Respondents’ knowledge about complication of COVID-19

COVID-19 disease was associated with untreated infections. Two hundred thirteen (54.3%) of the respondents stated that it could result in pneumonia and 279  (71.2%) in respiratory failure (Fig. 1).

### HCWs attitudes towards COVID-19 vaccines

The mean score of attitudes was 13.31, with a standard deviation of 1.529. The overall positive attitude rate about the COVID-19 vaccine was 52.3% with 95% CI (47.7 – 57.4%). Of those who had a positive attitude towards the COVID-19 vaccine, 189 (79.75%) were planning to get vaccinated when a vaccine becomes available. One hundred-nine (55.05%) males and 96 (49.5%), females had a positive attitude while 89 (44.95%) of males and 98 (50.5%) of females had a negative attitude towards COVID-19 vaccines.

Fifteen (44.12%) of the medical doctors, 30 (65.23%) laboratory technicians, 66 (50.77%) nurses and midwives, and 32 (56.14%) non-medical auxiliary staff had a positive attitude towards the COVID-19 vaccine. Of those who had a chronic disease, 19 (55.9%) had a positive attitude towards COVID-19 vaccines. Two-fifths (40.6%) of the HCWs were confident that the Ministry of Health can control COVID-19 in Ethiopia but 55.4% of them expressed a general mistrust/uncertainty about the effectiveness of COVID-19 vaccines (Table 3).

**Table 3:** Attitudes towards COVID-19 vaccines among healthcare workers in Dessie City, Ethiopia, May 2021

Questions	Frequency (*n*)	Percentage (*%*)
Are you confident that the Ministry of Health can control COVID-19 in Ethiopia? *		
Yes	159	40.6
No	233	59.4
Do you have general mistrust/uncertainty about COVID-19 vaccine effectiveness? *		
Yes	217	55.4
No	175	44.6
Do you fear COVID-19 vaccines? *		
Yes	221	56.4
No	171	43.6
**Reasons for fearing COVID-19 vaccines (*N*** **= 221)**		
Infertility	55	24.9
Severe allergic reactions	72	32.6
Unknown long-term effects	94	42.5
**Are you planning to be vaccinated for COVID-19?**		
Yes	251	64.0
No	141	36.0
Do you refuse to get vaccinated because only God/Allah can prevent COVID-19?*		
Yes	137	34.9
No	255	65.1
**What alternative preventive measures did you use to prevent COVID-19**		
Traditional medicine	15	3.8
Praying	48	12.2
Physical exercise	43	11.0
Should people with chronic and severe diseases get priority for COVID-19 vaccination? *		
Yes	341	87.0
No	51	13.0
Should HCWs get priority in COVID-19 vaccination? *		
Yes	340	86.7
No	52	13.3
Should all HCWs be vaccinated to protect the public? *		
Yes	276	70.4
No	62	15.8
I don't know ^δ^	54	13.8
To protect the public, HCWs should follow government guidelines about vaccines *		
Agree	322	82.1
Disagree	70	17.9

*These questions are used to compute the overall score of attitude

^δ^***To compute the overall score of Attitude, the third choice “I do not know” in some questions were included in the “No” category for analysis***

### Perceptions of HCWs about COVID-19 vaccines

The mean score of perceptions was 14.66, with a standard deviation of 1.866. The overall rate of good perception about the COVID-19 vaccine was 60.5% with 95% CI (55.6–65.3%). Of those who had a good perception about COVID-19 vaccine, only 189 (79.75%) were planning to be vaccinated. Nearly two-thirds 125 (63.13%) of the male and 112 (57.73%) of the female HCWs had a good perception whereas 73 (36.87%) of the males and 82 (42.3%) of the females had a poor perception of COVID-19 vaccines.

Nineteen (55.9%) doctors, 32 (69.57%) laboratory technicians, 86 (66.15%) nurses and midwives, 24 (48%) pharmacists, and 33 (57.9%) non-medical auxiliary staff had a good perception about COVID-19 vaccines. One hundred twenty-six (59.15%) of the Orthodox Christians, 16(55.17%) of the Protestants, and 95(63.3%) Muslims had a good perception about COVID-19 vaccines. Eighty-nine (58.9%), 44 (57.14%) and 104 (63.41%) HCWs who grew up in rural, semi-urban and urban areas, respectively, had a good perception of the COVID-19 vaccines. Among those who had a chronic disease, 23 (67.65%) had good perceptions.

Three-quarters (292, 74.5%) of the HCWs considered themselves to be at high risk of becoming infected with COVID-19 and 39.5% of them thought that they could get infected with COVID-19 through vaccination. Of these, 56 (14.3%) in the 20-30 age group, 67 (17.1%) aged 31-40, 26 (6.6%) aged 41-50 and 6 (1.5%) in the 51-60 age  group linked vaccines to COVID-19 infection. With regard to educational level, 6 (1.5%) HCWs with secondary education and below (grades 12 and below), 49 (12.5%) with diplomas, and 100 (25.5%) with university degrees associated vaccines with infection. Among the different occupational categories, 16 (4.1%) medical doctor, 21 (5.4%) medical laboratory technician, 50 (12.6%) nurse and midwive, 18 (4.6%) pharmacist,15 (3.8%) public health officer, 9 (2.3%) anesthesiologist, 10 (2.6%) radiographer and 16 (4.1%) non-medical auxiliary staff believed that they could get infected with COVID-19 through vaccination. Similarly, nearly half (184, 46.9%) of the HCWs thought that vaccines could worsen any pre-existing medical conditions. On the other hand, 173 (44.1%) of the respondents thought that it may not be possible to reduce the incidence of COVID-19 without vaccination (Table 4).

**Table 4:** Perception towards COVID-19 vaccine among healthcare workers in Dessie City, Ethiopia, May 2021

Questions	Frequency (*n*)	Percentage (*%*)
**Do you think you are at high risk of becoming infected with COVID-19?**		
Yes	292	74.5
No	73	18.6
I don’t know ^δ^	27	6.9
**Do you think that you could get infected with COVID-19 through vaccination?**		
Yes	155	39.5
No	165	42.1
I don’t know ^δ^	72	18.4
**Is it possible to reduce and control the incidence of covid-19 without vaccination?**		
Yes	173	44.1
No	174	44.4
I don’t know ^δ^	45	11.5
**Do most of my colleagues appear to think that getting vaccinated is a good idea?**		
Yes	272	69.4
No	72	18.4
I don’t know ^δ^	48	12.2
**Is getting yourself vaccinated for COVID-19 a good way to protect your family and other people against infection?**		
Yes	274	69.9
No	83	21.2
I don’t know ^δ^	35	8.9
**My family and friends think that getting vaccinated for COVID-19 is a good idea**		
Agree	285	72.7
Disagree	60	15.3
Neutral ^δ^	47	12.0
**Do you think that the COVID-19 vaccine can worsen any health conditions you have?**		
Yes	184	46.9
No	140	35.7
I don’t know ^δ^	68	17.4
**Do you believe that you can get proper medical care if you contract COVID-19?**		
Yes	197	50.3
No	131	33.4
I don’t know ^δ^	64	16.3
**Do you think that the development of COVID-19 vaccines was properly carried out to make them safe?**		
Yes	282	71.9
No	110	28.1

^δ^*These questions are included to compute the overall score of perception, the third choice “I don’t know/ neutral” in some questions was categorized into the “No/disagree” category for the analysis*

Nearly two-thirds 251 (64.0%) with 95%CI (59.4-68.6%) of the HCWs were planning to be vaccinated and 141 (36.0%) with 95% CI (31.4-40.6%) refused to be vaccinated for COVID-19 (Table 3). Bivariate analysis identified the following factors in the refusal to be vaccinated: absence of COVID-19 in the family or among colleagues and friends, failure to be tested for COVID-19, failure to obtain training or orientation about COVID-19 vaccination, negative attitude, and poor knowledge and perception   (Table 5).

**Table 5:** Bivariate analysis of factors associated with refusal of COVID-19 vaccine among healthcare workers in Dessie City, Ethiopia, May 2021

Variables	Refusal of vaccination		COR (95% CI)
	Yes	No	
**Sex**			
Male	68	130	0.87(0.57-1.31)
Female	73	121	Ref
**Age (years)**			
20-30	59	98	1.36(0.39-4.59)
31-40	57	99	1.29(0.38-4.39)
41-50	21	45	1.05(0.29-3.80)
51-60	4	9	Ref
**Educational status** *			
Secondary or below level	4	12	0.65(0.20-2.08)
Diploma	50	69	1.42(0.91-2.21)
University degree	87	170	Ref
**Religion** *			
Christian	96	146	1.53(0.99-2.37)
Muslim	45	105	Ref
**Marital status**			
Currently single	67	111	1.14(0.76-1.73)
Married	74	140	Ref
**Place of residence where you grew up**			
Rural	58	93	1.24(0.78-1.96)
Semi-urban	28	49	1.13(0.64-1.99)
Urban	55	109	Ref
**Household size (persons)**			
Five or less	103	174	1.19(0.76-1.89)
More than five	38	77	Ref
**Duration of work as HCW**			
10 years or less	114	192	1.29(0.78-2.16)
>10 years	27	59	Ref
**Have health insurance**			
No	117	198	1.31(0.77-2.23)
Yes	24	53	Ref
Anyone in your family or colleague or friend had COVID-19 *			
No	107	156	1.92(1.21-3.04)
Yes	34	95	Ref
Tested for COVID-19 *			
No	104	156	1.71(1.09-2.69)
Yes	37	95	Ref
**Have a chronic disease**			
No	130	228	1.19(0.56-2.52)
Yes	11	23	Ref
**Smoking status**			
Currently smoke	2	10	0.34(0.07-1.57)
Ex-smoker	10	23	0.74(0.34-1.59)
Never smoked	129	218	Ref
Profession *			
Medical doctor	15	19	Ref
Laboratory technician	16	30	0.68(0.27-1.68)
Nurse and midwive	32	98	0.41(0.19-.91)
Pharmacist	29	21	1.75(0.73-4.22)
Radiographer	7	11	0.81(0.25-2.58)
Anesthesiologist	10	11	1.15(0.39-3.43)
Public health officer	8	28	0.36(0.13-1.02)
Non-medical auxiliary staff	24	33	0.92(0.39-2.17)
Received training or orientation about COVID-19 vaccination *			
No	99	136	1.99(1.29-3.09)
Yes	42	115	Ref
Knowledge level about COVID-19 vaccine*			
Poor	67	80	1.94(1.27-2.96)
Good	74	171	Ref
Attitude towards COVID-19 vaccine*			
Negative	97	90	3.94(2.54-6.12)
Positive	44	161	Ref
Perceptions of COVID-19 vaccine*			
Poor	93	62	5.91(3.76-9.27)
Good	48	189	Ref

*Indicates variables included in the multivariable logistic regression analysis; Ref, reference category

### Proportion of refusal to be vaccinated for COVID-19

One hundred forty-one (36.0%, 95% CI [31.4-40.6%]) refused to be vaccinated for COVID-19 when vaccines become available and 251 (64.0%, 95% CI [59.4- 68.6%]) said that they wanted to be vaccinated.

### Factors associated with refusal to be vaccinated

In multivariable analysis, refusal to be vaccinated for COVID-19 vaccine was significantly associated with a negative attitude (AOR: 3.06; 95% CI [1.86-5.03]) and poor perception (AOR: 4.73; 95% CI [2.91-7.68]). HCWs who had negative attitude about COVID-19 vaccines were 3.06 times refused to be vaccinated compared to those who had positive attitude towards COVID-19 vaccines. Furthermore, HCWs who had poor perception about COVID-19 vaccines were 4.73 times refused to be vaccinated compared to those who had good perception towards COVID-19 vaccines.  However, being nurses and midwives (AOR: 0.38; 95% CI [0.15-0.97]) was significantly associated with to be vaccinated for COVID-19 vaccine compared to other HCWs. Also, most pharmacists refused to be vaccinated but this association was not statistically significant (Table 6).

**Table 6:** Multivariable analysis of factors significantly associated with refusal to be vaccinated for COVID-19 vaccine among healthcare workers in Dessie City, Ethiopia, May 2021

Variables	Refusal of vaccinations		COR (95% CI)	AOR (95% CI)	*P-value* (adjusted model)
	Yes	No			
**Profession**					
Medical doctor	15	19	Ref	Ref	
Laboratory technician	16	30	0.68(0.27-1.68)	0.98(0.34-2.83)	0.973
Nurse and midwive	32	98	0.41(0.19-.91)	0.38(0.15-.97)	0.042
Pharmacist	29	21	1.75(0.73-4.22)	1.50(0.53-4.25)	0.445
Radiographer	7	11	0.81(0.25-2.58)	0.66(0.16-2.33)	0.470
Anesthesiologist	10	11	1.15(0.39-3.43)	0.72(0.20-2.60)	0.617
Public health officer	8	28	0.36(0.13-1.02)	0.46(0.14-1.50)	0.197
Non-medical auxiliary staff	24	33	0.92(0.39-2.17)	0.97(0.36-2.66)	0.955
**Attitude towards COVID-19 vaccine**					
Negative	97	90	3.94(2.54-6.12)	3.06(1.86-5.03)	< 0.001
Positive	44	161	Ref	Ref	
**Perception of COVID-19 vaccine**					
Poor	93	62	5.91(3.76-9.27)	4.73(2.91-7.68)	< 0.001
Good	48	189	Ref	Ref	

## Discussion

On-going efforts are  been made to end the COVID-19 pandemic. Various COVID-19 vaccines have been distributed in many countries, including Ethiopia. The overall good knowledge, good perception, and positive attitude rates of the HCWs about COVID-19 vaccines were 62.5%, 60.5%, and 52.3%, respectively.

In our study, 64.0% of the HCWs planned to be vaccinated and 36.0% refused to do so. A similar vaccine acceptance rate was reported by a study in Iraq (61.7%) [18], which was higher than in two studies in the USA, where more than half of all HCWs were undecided and delayed the decision to be vaccinated [19, 20]. Low acceptance rates were also reported among healthcare workers in Ghana (39.3%) [21]), the Democratic Republic of Congo (27.7%) [22]), Egypt (21%) [23]), and Nepal (38.3%) [24]). Studies in Nigeria and Saudi Arabia reported intended vaccine uptake rates of 50.2% [25] and 50.52%, respectively [26]. Low rates may be due to earlier study dates (when prospects of the vaccine rollout were uncertain), HCWs limited knowledge about vaccines, lack of trust in government management capacity, and concerns about vaccine safety.

Some of the highest vaccine acceptance rates (above 95%) were reported from South and Southeast Asia, where HCWs were willing to be vaccinated because as they perceived the pandemic to be severe, considered the vaccines to be safe, experienced few financial constraints and little stigmatization of being vaccinated, and trusted the health authorities [27]. Studies in China and Vietnam reported intended vaccine uptakes of 76.63% [28] and 76.10%, respectively [29], slightly higher than our findings. These high rates were associated with good knowledge regarding the severity of COVID-19, HCWs’ trust in the vaccines, and earlier study dates than in our study.

In this study, high rates of not intending to be vaccinated when vaccines become available were associated with negative attitude and low perception about COVID-19 vaccine. Our study also showed that being a nurse and midwife was significantly associated with acceptance to be vaccinated for COVID-19. Consistent with our findings, a study in India found vaccine acceptance to be highest among nurses [30]. However, in a study in the Democratic Republic of the Congo, the highest acceptance rates among HCWs were reported for medical doctors [22] and in Debre Tabor Hospital in northern Ethiopia, nurses had the lowest acceptance rates [31].

Our findings corroborate a study from the Democratic Republic of the Congo that reported that having a positive attitude towards COVID-19 vaccines was significantly associated with the willingness to receive a vaccine [22]. A study among HCWs of an inner-city hospital in New York [32] reported similar results. A study in Libya indicated that having a family member or friend infected with COVID-19 was positively associated with the likelihood of vaccine acceptance [33]. A community-based study in Wolaita Zone, Ethiopia, showed that family members and friends who had been tested for COVID-19 were significantly associated with the acceptance of COVID-19 vaccine [34]. In our study, having COVID-19-infected family members, colleagues, or friends was associated with increased vaccine uptake intention only in the bivariate analysis but not in the final adjusted logistic model (Table 5). In addition, as indicated in Table 4, 39.5% of the HCWs associated vaccines with possible COVID-19 infections and 46.9% of them thought that vaccines could worsen any pre-existing medical conditions. This is very concerning, as it indicates that HCWs are not convinced about the safety of COVID-19 vaccines and are also misinformed. The reason for this widespread misinformation is not known but the public health agencies in Ethiopia may need to increase their efforts to educate HCWs about the safety of these vaccines.

### Limitation of the study

Bias may have affected the result of our study. Social desirability bias in which HCWs answered questions in a manner that would be viewed favorably by others may have resulted in over-reporting of good attitudes and perceptions as well as intended uptake of vaccines. Moreover, causal inferences cannot be drawn from this study due to the nature of the study design.

## Conclusion

The willingness to get vaccinated against COVID-19 was moderately high among healthcare workers in Dessie City. Negative attitudes and poor perceptions of HCWs towards COVID-19 vaccines were the most significant factors in the refusal to accept vaccines. Because HCWs are scheduled to be among the first to receive vaccines and play a central role in their administration in the population, key factors in their decision-making process, such as knowledge about the safety of vaccines, must be addressed as early as possible. Our findings may inform health planners and administrators in developing relevant interventions that promote COVID-19 vaccination uptake among healthcare workers in Ethiopia. In particular, the large proportions of HCWs who considered vaccines to worsen any pre-existing medical conditions or cause COVID-19 is very concerning, as it seems that HCWs are not convinced about the safety of the vaccines and are also misinformed. These issues need to be urgently addressed by the public health agencies in Ethiopia. Working on behavioral change communication and social mobilization towards COVID-19 vaccines using culturally appropriate manner might increase the attitudes and perceptions, which in turn increasing the rate of COVID-19 vaccination. We also recommend studies in urban and rural communities to investigate the religious conspiracy about COVID-19 vaccines  that may ensure increasing the vaccination rate of COVID-19.

### Implication of the study

The information on attitudes, knowledge and perceptions pertaining to the refusal of COVID-19 vaccine among HCWs can inform policy makers and administrators about opportunities and constraints in distributing vaccines among HCWs in Dessie and other Ethiopian cites. The findings may thus contribute to developing a strategy for controlling the pandemic by addressing factors significantly affecting vaccination uptake.

## Data Availability

The datasets analyzed during the current study are available from the corresponding author on reasonable request.

## Abbreviations

AOR: adjusted odds ratio
CI: confidence interval
COR: crude odds ratio
HCWs: healthcare workers
